# 

**Published:** 1909-12

**Authors:** 


### The Library of the Bristol Medico-Chirurgical Society.

*The following donations have been received since the publication of the List in September.*

*November 30th*, 1909.

| | |
|---|---|
| George M. Gould, M.D. (1) .. .. .. .. | 1 volume. |
| L. M. Griffiths .. .. .. .. .. | 19 volumes. |
| R. Shingleton Smith, M.D. (2) .. .. .. | 6 ,, |

### SEVENTY-FOURTH LIST OF BOOKS.

The titles of books mentioned in previous lists are not repeated.

The figures in brackets refer to the figures after the names of the donors, and show by whom the volumes were presented. The books to which no such figures are attached have either been bought from the Library Fund, or received through the *Journal*.

**Allbutt and H. D. Rolleston, Sir C.** (Eds.) *A System of Medicine.* Vol. VI .. .. .. .. .. .. .. .. .. 1909

**Basu, B. D.** .. .. *The Dietetic Treatment of Diabetes* .. .. .. .. 1909

**Branson, H. Thursfield and W. P. S.** *Medical Morbid Anatomy and Pathology* .. .. .. .. .. .. .. .. 1909

**Burghard, F. F.** (Ed.) *A System of Operative Surgery.* Vols. II, III 1909

*Chemistry* (Catechism Series) .. .. .. .. .. .. .. .. .. .. .. [N.D.]

**Clarke, H.** .. .. *Studies in Tuberculosis* .. .. .. .. .. .. [1909]

**Clay, Rotha M.** .. *The Mediæval Hospitals of England* .. .. [1909]

*Deuteronomy Smith* .. .. .. .. .. .. .. .. .. .. .. .. .. .. .. [N.D.]

**Downie, J. W.** .. *Diseases of the Throat* .. .. .. 2nd Ed. 1909

**Glaister, J.** .. .. *Arsenical Gas Poisoning* .. .. .. .. .. .. 1908

**Gould, G. M.** .. .. *Biographic Clinics* .. .. .. (1) Vol. VI 1909

**Greer, W. J.** .. .. *Industrial Diseases and Accidents* .. .. .. 1909

**Haberston, S. H.** .. *Diseases of the Stomach* .. .. .. .. .. .. 1909

**Herschell, G.** .. *Soured Milk and Pure Cultures of Lactic Acid in the Treatment of Disease* .. .. 2nd Ed. 1909

**Jennings, O.** .. .. *The Morphia Habit* .. .. .. .. .. .. .. .. 1909

**Keirle, N. G.** .. .. *Studies in Rabies* .. .. .. .. .. .. .. .. 1909

**Lejars, F.** .. .. *Urgent Surgery.* (Tr. from 6th German Edition by W. S. Dickie) .. .. Vol. I 1909

**Magennis, E.** .. *Dictionary of Ophthalmic Terms* .. .. .. [1909]

**Marie, A.** (Ed.) .. *Traité international de Psychologie pathologique* Tome I 1910

**Murphy, D'A. Power and J. K.** (Eds.) *A System of Syphilis* Vol. III 1909

**Osler, W.** .. .. *An Alabama Student, and other Essays* .. .. 1908

**Pottenger, F. M.** .. *Pulmonary Tuberculosis* .. .. .. .. .. (2) 1908

**Power and J. K. Murphy, D'A.** (Eds.). *A System of Syphilis* Vol. III 1909

**Ranney, A. L.** .. *Lectures on Nervous Diseases* .. .. .. .. (2) 1889

**Rolleston, Sir C. Allbutt and H. D.** (Eds.) *A System of Medicine* Vol. VI 1909
**Rotter** [ ].. .. *Typische Operationen* (Ed. by A. Schönwerth) 8th Ed. 1909
**Sainsbury, H.** .. *Principia Therapeutica* .. .. .. .. .. .. [1906]
**Savill, T. D.** .. .. *A System of Clinical Medicine* .. .. 2nd Ed. 1909
**Strauss, H.** .. .. *Gout* .. .. .. .. .. .. .. .. .. .. .. .. 1909
**Thursfield and W. P. S. Branson, H.** *Medical Morbid Anatomy and Pathology* .. .. .. .. .. .. .. 1909
**Walters, F. R.** .. *Sanatorium Treatment of Pulmonary Tuberculosis* .. .. .. .. .. .. .. .. .. .. 1909

### TRANSACTIONS, REPORTS, JOURNALS, &c.

American Dermatological Association, Transactions of the .. .. 1908

American Journal of Obstetrics, The .. .. .. .. Vol. LIX 1909

American Journal of Medical Sciences .. .. .. Vol. CXXXVII 1909

American Urological Association, Transactions of the .. Vol. II 1909

Boston Medical and Surgical Journal, The .. .. .. Vol. CLX 1909

Edinburgh Medical Journal, The .. .. .. .. .. .. N.S., Vol. II 1909

Edinburgh Obstetrical Transactions .. .. .. .. Vol. XXXIV 1909

Glasgow Medical Journal, The .. .. .. .. .. .. Vol. LXXI 1909

Hospital, The .. .. .. .. .. .. .. .. .. .. .. .. .. Vol. XLV 1909

Hospitals—

Philadelphia General Hospital Reports.. .. .. Vol. VII 1909

Westminster Hospital Reports, The .. .. .. Vol. XVI 1909

Journal of Medical Research, The .. .. .. .. .. .. .. Vol. XX 1909

Journal of Obstetrics and Gynæcology, The.. .. .. Vol. XV 1909

Journal of the American Medical Association, The .. Vol. LII 1909

Medical Record.. .. .. .. .. .. .. .. .. .. .. .. .. Vol. LXXV 1909

Münchener medizinische Wochenschrift .. .. Bd. I für 1909

Practitioner, The .. .. .. .. .. .. .. .. .. .. Vol. LXXXII 1909

Progrès médical, Index du .. .. .. .. .. .. .. .. .. .. .. .. 1909

Surgery, Gynecology and Obstetrics .. .. .. .. Vol. VIII 1909

Univ. of Penna. Medical Bulletin .. .. .. .. .. .. Vol. XXI 1909

### MEETINGS OF SOCIETIES.

### Bristol Medico-Chirurgical Society.

### Local Medical Notes.

### BRISTOL.

**University of Bristol.**—FACULTY OF MEDICINE :—

EXAMINATION SUCCESSES.

UNIVERSITY OF LONDON.—In the M.B., B.S. Examination, J. W. J. Wilcox was successful in the Honour's Division, and obtained distinction in Medicine.

M.D. Examination.—The University Medal was awarded to Henry Devine for Mental Diseases, not as stated in our last issue for Diseases of Women.

CONJOINT EXAMINING BOARD OF ENGLAND.—*Materia Medica :* B. C. Eskell, H. R. B. Hull. *Anatomy and Physiology :* E. S. Abraham. *Medicine :* P. J. Martin,* F. C. Morgan.* *Surgery :* H. H. S. Templeton,* J. F. H. Morgan.* *Midwifery :* J. F. H. Morgan,* M. M. Lopez. *R.A.M.C. :* P. C. Field, M.R.C.S.

A students' reception was held on October 2nd, and was a decided success. The students were received by Lady Owen, Professor and Mrs. Lloyd Morgan, and Professor and Mrs. Michell Clarke. Music was provided by the University Musical Society.

An interesting exhibit of Antarctic objects was shown by Mr. R. E. Priestley, who was geologist to Sir Ernest Shackleton's expedition. Amongst the refreshments was tea which had been buried in the ice for about eight years, and was brought back by this expedition. Its taste had been little impaired by its long stay in the ice.

* Denotes qualification.

A generous donor to the University funds, Mr. Henry Overton Wills, has, we are pleased to note, received the Freedom of the City of Bristol, an honour held by few.

**Bristol Medical Dinner.**—The Annual Dinner, held on November 23rd, under the presidency of Dr. Herapath, was very successful, being attended by a large number of past as well as present students. The Vice-Chancellor of the University was the very popular guest of the evening.

**Bristol General Hospital.**—At the half-yearly meeting of the subscribers of this institution, the financial statement showed that the deficit amounted to £3,000. The medical report was satisfactory, and showed the great need of such an institution in the locality.

The following appointments have recently been made: *House Surgeon:* F. C. Morgan, M.R.C.S., L.R.C.P. *House Physician:* T. B. Dixon, M.R.C.S., L.R.C.P. *Assistant House Physician:* J. F. H. Morgan, M.R.C.S., L.R.C.P. *Casualty Officer:* D. Robertson, M.B., B.Ch.Edin.

### APPOINTMENTS.

W. M. Bergin, M.B., B.S.Lond., has been appointed Assistant-Surgeon to the Royal Eye Hospital, Southwark.

P. S. Connellan, M.B., B.S., has been appointed House Surgeon to the Gordon Hospital, London.

Forbes Fraser, F.R.C.S.Eng., has been appointed an Honorary Surgeon to the Royal United Hospital, Bath.

**Declining Birth Rate in Bristol.**—It was stated at a recent meeting of the Health Committee that whereas in 1884 the birth-rate was 32.6, a year ago it was 24.2, and now it was as low as 23.

### BERKELEY.

**Jenner Tribute.**—A visit was paid recently by Dr. Simon Shiwopiszew, Director of the Russian Lymph Institution at Orel, and Lecturer on Vaccination, and M. Edward Hoffmann, of Finland, to Berkeley, to place a wreath on Jenner's tomb in the chancel of the parish church. They were afterwards shown a collection of Jenner relics by a local collector, with which they expressed keen delight.

### GLOUCESTER.

**Royal Infirmary.**—At a recent quarterly meeting of the governors, the chairman stated that the expenditure last year exceeded the income by £1,000. Unless they had increased support one of the wards would be closed, which would be a calamity to the city and county; but the increasing cost made it necessary for more generous donations to be made to carry on the work of the Infirmary successfully

### INDEX.

Alexander, Dr. D. A.—Cretinism and the puerperium, 128.
Almond, Dr. G. H.-H.—Theoretical considerations of pulmonary percussion notes, 215.
Anæsthesia, The safety of the patient in—Dr. J. Freeman, 238.
Anæsthesia, Vomiting connected with—A. L. Flemming, 40.
Anaphylaxis, 155.
Antitoxin treatment in diphtheria, 287.
Aphasia, 152.
Appendicostomy, 67.

Bacteria, Sensitisation of, 156.
Bárány's tests, 70.
Blood pressure, Measurement of, 341.
**Books, Reviews of—**
- Allbutt and Dr. H. D. Rolleston, Sir C. [Eds.]—A system of medicine, 71, 353.
- American Pediatric Society, Transactions of the, 363.
- American Proctologic Society, Transactions of the, 264.
- Appendicitis ? Has surgical treatment lessened the mortality from—Dr. F. P. Vale, 165.
- Arteries, Suture of—Dr. A. E. Smith, 358.
- Association of American Physicians, Transactions of the, 260.
- Atkinson, Dr. S. B.—The law in general practice, 176.
- Babies, Lectures on—Dr. R. Vincent, 76.
- Baby, Our—Mrs. J. L. Hewer, 75.
- Bacteria, Differential diagnosis of —Dr. E. P. Minett, 362.
- Bailey, Dr. P. J.—The care and nursing of the insane, 176.
- Ball, Sir C. B.—The rectum: its diseases and developmental defects, 167.
- Barnett, H. N.—Legal responsibility of the drunkard, 173.
- Bennett, Sir W.—Injuries and diseases of the knee-joint, 263.

Books, Reviews of (*continued*) —
- Bennett, Sir W. H.—Massage in recent fractures, 361.
- Bland-Sutton, J.—Essays on the position of abdominal hysterectomy in London, 264.
- Bland-Sutton and Dr. A. E. Giles, J.—The diseases of women, 264.
- Blood pressure, Studies in—Dr. G. Oliver, 73.
- Brown, Dr. W. L.—Physiological principles in treatment, 247.
- Brunton and L. Rogers, Sir J. Fayrer, Sir L.—On the poison of venomous snakes, 350.
- Bunge, Dr. G. v.—Text-book of organic chemistry for medical students, 268.
- Burghard, F. F. [Ed.]—A system of operative surgery, 249, 355.
- Burton-Fanning, Dr. F. W.—The open-air treatment of pulmonary tuberculosis, 365.
- Cancer, Pathology of—Dr. C. P. White, 267.
- Cardiac examination, Synoptic chart of, 361.
- Catalogue (Index-) of the library of the Surgeon-General's office, United States Army, 177.
- Chemistry, Organic—Dr. G. v. Bunge, 268.
- Childe, C. P.—Operative nursing and technique, 360.
- Children, Guide to the clinical examination and treatment of sick—Dr. J. Thomson, 174.
- Clark, Drs. D. N. Paton and G. H. —A practical source of general physiology, 173.
- Clinical methods — Drs. R. Hutchison and H. Rainy, 73.
- Compton, A. T.—Essentials of surgery, 175.
- Crichton - Browne, Sir J.— Parcimony in nutrition, 362.
- Death and its verification—J. B. James, 176.
- Dental anatomy, Notes on— G. A. Peake, 268.

Books, Reviews of (*continued*)—
Dental surgery, Golden rules of—C. W. Glassington, 364.
Dermatology, Introduction to—Dr. N. Walker, 74.
Diet and dietetics, A system of—Dr. G. A. Sutherland [Ed.], 256.
Diet in infancy—Dr. A. D. Fordyce, 257.
Downie, Dr. J. W.—Clinical manual of diseases of the throat, 367.
Drunkard, Legal responsibility of—H. N. Barnett, 173.
Ears, Infected—F. F. White, 366.
Encyclopædia and dictionary of medicine and surgery, 174, 259.
Eye, Diseases of the—Dr. C. H. May and C. Worth, 265; M. S. Mayou, 266.
Fayrer, Sir L. Brunton and L. Rogers, Sir J.—On the poison of venomous snakes, 350.
Fordyce, Dr. A. D.—Diet in infancy, 257.
Forel, Dr. A.—Hypnotism, 169.
Fowler, Dr. J. S.—Infant feeding, 257.
Fractures, Massage in recent—Sir W. H. Bennett, 361.
Freyer, Dr. P. J.—Clinical lectures on the surgical diseases of the urinary organs, 357.
Giles, J. Bland-Sutton and Dr. A. E.—Diseases of women, 264.
Glassington, C. W.—Golden rules of dental surgery, 364.
Goodall and J. W. Washbourn, Drs. E. W.—A manual of infectious diseases, 266.
Gresswell, G. and A.—Health, morals and longevity, 360.
Hay, Dr. J.—Graphic methods in heart disease, 349.
Hay fever, hay asthma—W. Lloyd, 175.
Health, morals and longevity—G. and A. Gresswell, 360.
Heart, Diseases of the—Dr. J. Mackenzie, 248.
Heart disease, Graphic methods in—Dr. J. Hay, 349.
Heart, The "Nauheim" treatment of diseases of the—Dr. L. T. Thorne, 359.
Heredity on disease, Influence of, 354.
Hewer, Mrs. J. L.—Our baby, 75.

Books, Reviews of (*continued*)—
Hogarth, Dr. A. H.—Medical inspection of schools, 351.
Hood, Dr. D. W. C.—Some of the clinical aspects of pneumonia, 77.
Hopf, L.—The human species, 349.
Howell, Drs. H. W. Wilson and C. M. H.—Movable kidney, 358.
Hutchinson, Sir F. Treves and J.—A manual of operative surgery, 361.
Hutchison and H. Rainy, Drs. R.—Clinical methods, 73.
Hygiene and public health—Drs. B. A. Whitelegge and G. A. Newman, 266.
Hypnotism—Dr. A. Forel, 169.
Hysterectomy in London, Essays on the position of abdominal—J. Bland-Sutton, 264.
Immunity, Studies on—Dr. R. Muir, 362.
Infancy, Diet in—Dr. A. D. Fordyce, 257.
Infant feeding—Dr. J. S. Fowler, 257.
Infectious diseases, Manual of—Drs. E. W. Goodall and J. W. Washbourn, 266.
Infectious diseases, Practical textbook of—Dr. R. W. Marsden, 177.
Insane, Care and nursing of the—Dr. P. J. Bailey, 176.
Insane, Handbook for attendants on the, 268.
Ions and their use in medicine, Electric—S. Leduc, 74.
Ireland, Transactions of the Royal Academy of Medicine in, 367.
James, J. B.—Death and its verification, 176.
Kelynack, Dr. T. N. [Ed.]—Tuberculosis in infancy and childhood, 72.
Kidney, Movable—Dr. D. Newman, 253; Drs. H. W. Wilson and C. M. H. Howell, 358.
Knee-joint, Injuries and diseases of the—Sir W. Bennett, 263.
Law in general practice, The—Dr. S. B. Atkinson, 176.
Leduc, S.—Electric ions and their use in medicine, 74.
Lendon, Dr. A. A.—Nodal fever, 77.
Liver, Common affections of the—Dr. W. H. White, 174.

Books, Reviews of (*continued*)—
Lloyd W.—Hay fever, hay asthma, 175.
Macdonald, I.—Home nursing, 364.
MacIlwain, S. W.—The future of medicine, 259.
Mackenzie, Dr. J.—Diseases of the heart, 248.
Marsden, Dr. R. W.—A practical text-book of infectious diseases, 177.
Marshall, Dr. C. F.—Golden rules of venereal disease, 175.
Martindale and Westcott—The extra pharmacopœia, 177.
Massage in recent fractures—Sir W. H. Bennett, 361.
May and C. Worth, Dr. C. H.—Diseases of the eye, 265.
Mayou, M. S.—Diseases of the eye, 266.
Medical annual, The, 266.
Medicine, A system of—Sir C. Allbutt and Dr. H. D. Rolleston [Eds.], 71, 353.
Medicine, The future of—S. W. MacIlwain, 259.
Medico-Chirurgical Society of Edinburgh, Transactions of the, 261.
Medico-Chirurgical transactions, 259.
Mental deficiency—A. F. Tredgold, 171.
Merck's annual report, 366.
Mind and its disorders — Dr. W. H. Stoddart, 254.
Minett, Dr. E. P.—Differential diagnosis of bacteria, 362.
Muir, Dr. R.—Studies on immunity, 362.
Murphy, Drs. D'A. Power and J. K. [Eds.]—A system of syphilis, 251.
Nauheim treatment of diseases of the heart—Dr. L. T. Thorne, 359.
Nursing, Home—I. Macdonald, 364.
Nutrition, Parcimony in—Sir J. Crichton-Browne, 362.
Newman, Dr. D.—Movable kidney, and other displacements and malformations, 253.
Newman, Drs. B. A. Whitelegge and G.—Hygiene and public health, 266.
Nodal fever—Dr. A. A. Lendon, 77.

Books, Reviews of (*continued*)—
Oliver, Dr. G.—Studies in blood pressure, 73.
Open-air treatment of pulmonary tuberculosis — Dr. F. W. Burton-Fanning, 365.
Operative nursing and technique —C. P. Childe, 360.
"Ophthalmoscope," The, 175.
Parents, teachers and schools—Dr. J. O. Symes, 363.
Paton, Dr. D. N.—Essentials of human physiology, 247.
Paton and G. H. Clark, Drs. D. N.—A practical course of general physiology, 173.
Peake, G. A.—Notes on dental anatomy, 268.
Pharmacopœia, The extra — Martindale and Westcott, 177.
Phillips, Dr. J.—Outlines of diseases of women, 265.
Physical signs of diseases of the thorax and abdomen—Dr. J. E. H. Sawyer, 73.
Physiological principles in treatment — Dr. W. L. Brown, 247.
Physiology, A practical course of general—Drs. D. N. Paton and G. H. Clark, 173.
Physiology, Essentials of human Dr. D. N. Paton, 247.
Physique of boys, The—Dr. B. M. H. Rogers, 27.
Pneumonia, Some of the clinical aspects of—Dr. D. W. C. Hood, 77.
Power, and J. K. Murphy, Drs. D'A.—A system of syphilis, 251.
Pulmonary tuberculosis, Open-air treatment of—Dr. F. W. Burton-Fanning, 365.
Pye's surgical handicraft, 361.
Rainy, Drs. R. Hutchison and R.—Clinical methods, 73.
Rectum, The: its diseases and developmental defects—Sir C. B. Ball, 167.
Remedies, New and non-official, 365.
Rogers, Sir J. Fayrer, Sir L. Brunton and L.—On the poison of venomous snakes, 350.
Rolleston, Sir C. Allbutt and Dr. T. C. [Eds.]—A system of medicine, 71, 353.
Saint Bartholomew's Hospital reports, 261.

Books, Reviews of (*continued*)—
Sawyer, Dr. J. E. H.—Physical signs of diseases of the thorax and abdomen, 73.
Schools, Medical inspection of——Dr. A. H. Hogarth, 351.
Smith, Dr. E. A.—Suture of arteries, 358.
Smithsonian institution reports, 76, 268.
Snakes, On the poison of venomous—Sir J. Fayrer, Sir L. Brunton and L. Rogers, 350.
Society for the study of disease in children, Reports of the, 75.
Species, The human—L. Hopf, 349.
Stoddart, Dr. W. H.—Mind and its disorders, 254.
Surgery, Essentials of—A. T. Compton, 175.
Surgery, Operative, A system of—F. F. Burghard [Ed.], 249, 355; Manual of—H. J. Waring, 262; Sir F. Treves and J. Hutchinson, 361.
Surgical handicraft, Pye's, 361.
Sutherland, Dr. G. A. [Ed.]—A system of diet and dietetics, 256.
Symes, Dr. J. O.—Parents, teachers and schools, 363.
Syphilis, A system of—Drs. D'A. Power and J. K. Murphy [Eds.], 251.
Thomson, Dr. J.—Guide to the clinical examination and treatment of sick children, 174.
Thorne, Dr. L. T.—The "Nauheim" treatment of diseases of the heart, 359.
Throat, Diseases of the—Dr. J. W. Downie, 367.
Treatment, Manual of medical—Dr. I. B. Yeo, 359.
Treatment, Physiological principles in—Dr. W. L. Brown, 247.
Tredgold, A. F.—Mental deficiency, 171.
Treves and J. Hutchinson, Sir F.—A manual of operative surgery, 361.
Tuberculosis in infancy and childhood—Dr. T. N. Kelynack [Ed.], 72.
Urinary organs, Clinical lectures on the surgical diseases of the—Dr. P. J. Freyer, 357.

Books, Reviews of (*continued*)—
Vale, Dr. F. P.—Has surgical treatment lessened the mortality from appendicitis? 165.
Venereal disease, Golden rules of—Dr. C. F. Marshall, 175.
Vincent, Dr. R.—Lectures on babies, 76.
Walker, Dr. N.—Introduction to dermatology, 74.
Waring, H. J.—Manual of operative surgery, 262.
Washbourn, Drs. E. W. Goodall and J. W.—A manual of infectious diseases, 266.
White Dr. C. P.—Lectures on the pathology of cancer, 267.
White, Dr. W. H.—Common affections of the liver, 174.
White, F. F.—Infected ears, 366.
Whitelegge and G. Newman, Drs. B. A.—Hygiene and public health, 266.
Wilson and C. M. H. Howell, Drs. H. W.—Movable kidney, 358.
Women, The diseases of—J. Bland-Sutton and Dr. A. E. Giles, 264; Dr. J. Phillips, 265.
Worth, Dr. C. H. May and C.—Diseases of the eye, 265.
Yeo, Dr. I. B.—A manual of medical treatment, 359.
Boys, The physique of—Dr. B. M. H. Rogers, 27.
Bristol and neighbourhood, Small-pox in—Dr. D. S. Davies, 97.
Bristol health committee, 371.
Bristol Medico-Chirurgical Society, 91, 186.
British Medical Association, Notes on the Belfast meeting, 269.

Calcium metabolism, tetany and the parathyroids, Relation between, 240.
Cardiac pain, Referred—Dr. F. G. Thomson, 193.
Carwardine, T.—The indications for gastro-enterostomy, 107.
Cataphoresis, Discussion on treatment by, 132.
Cerebral paralyses, The causes of transient—Dr. G. Parker, 15.
Cerebro-spinal fluid in some dermatoses of infancy, 164.
Charles, Dr. J. R.—Report on medicine, 240.

Christian science and faith healing, 368.
Clarke, Dr. J. M.—Precautions to be observed in administration of milk in disease, 57; Report on medicine, 152.
Colitis, Surgical treatment of, 67.
Colon, Excision of malignant growths of, 64; Functional diseases of, 65; The lymphatics of, 64; The movements of the colon and its contents, 63; Tuberculosis of—E. W. H. Groves, 203.
Convalescent Home, The Queen Victoria Jubilee, 80.
Cretinism and the puerperium—Dr. D. A. Alexander, 128.

Davies, Dr. D. S.—Milk from a public health standpoint, 44; Small-pox in Bristol and neighbourhood, 97.
Dermatology, Report on—Dr. J. A. Nixon, 161.
Diabetes, Treatment of, 342.
Diphtheria, Antitoxin treatment in, 287.
Dover, Thomas—Dr. J. A. Nixon, 31.

Editorial notes, 78, 178, 269, 368.
Electricity in general practice—Dr. H. P. Tayler, 145.
Endocarditis, Infectious or malignant, 339.
Exophthalmic goitre, Surgical treatment of, 243.
Eye, On metastatic inflammations of the—Dr. J. H. Parsons, 1.

Faure, Dr. M.—The mercurial treatment of tabes dorsalis, 312.
Fells, Dr. A.—Cases of pneumonia, 336.
Flemming, A. L.—Vomiting connected with anæsthesia, 40.
Fortescue-Brickdale, Dr. J. M.—Sleep and the modern hypnotics, 230; The heating of milk as it affects the nutrition and health of the infant, 61.
Fox (Long) Lecture, The, on metastatic inflammations of the eye—Dr. J. H. Parsons, 1.
Freeman, Dr. J.—The safety of the patient in anæsthesia, 238.
Fronto-ethmoidal sinus suppuration, Radical osteoplastic operation for double, 382.

Gastro-enterostomy, The indications for—T. Carwardine, 107.
Goitre, Exophthalmic, Surgical treatment of, 243.
Gravid uterus, Retroversion of the, 380.
Groves, E. W. H.—Report on surgery, 62; Tuberculosis of the colon, 203.

Hall, Dr. I. W.—Some impurities of vended milks, 48.
Hall and W. A. Smith, Drs. I. W.—The value of some lactic acid ferments, 56.
Heart, Wounds of the, 346.
Hernia *en masse*, Reduction of, 160; Inguinal in children, 158; Radical cure of, 157.
History and practice of surgery—Dr. J. Swain, 289.
Hypnotics, Sleep and the modern—Dr. J. M. Fortescue-Brickdale, 230.

Ileo-cæcal valve, its importance in surgery, 62.
Ileo-colostomy, Three cases of, 91.
Incurables, home for, Desirability of, 81.
International medical congress at Budapest, 372.
Iodoform and thyroidism, 382.
Ionisation, Discussion on treatment by, 132.
Intestine, Surgery of the large, 62.

James, J. A.—A case of lead encephalopathy, 124.
Joint swellings associated with enlargement of lymphatic glands, 188.
Jones, Dr. H. L.—Contribution to discussion on treatment by ionisation or cataphoresis, 138.

Kyle, H. G.—Report on surgery, 346.

Lactic acid ferments, The value of some—Drs. I. W. Hall and W. A. Smith, 56.
Laryngology, Report on—Dr. P. W. Williams, 69.
Lead encephalopathy, Case of—J. A. James, 124.
Library Association, British Medical, 95.

Library of the Bristol Medico-Chirurgical Society, 88, 184, 282, 377; Librarian's annual report, 379.
Local medical notes, 93, 189, 284, 383.
Lymphatic glands, Joint swellings associated with enlargement of, 188.
Lymphatics of the colon, 64.

Medicine, Reports on—Dr. J. R. Charles, 240; Dr. J. M. Clarke, 152; Dr. G. Parker, 339.
Meningitis, Recovery from tuberculous, 381.
Mercurial treatment of tabes dorsalis—Dr. M. Faure, 312.
Milk—Adulteration of, in Bristol—E. Russell, 47; From a public health standpoint, city of Bristol—Dr. D. S. Davies, 44; Heating of, as it affects the nutrition and health of the infant—Dr. J. M. Fortescue-Brickdale, 61; Impressionist report on, 83; In disease, Precautions to be observed in administration of—Dr. J. M. Clarke, 57.
Milks, Some impurities of vended—Dr. I. W. Hall, 48.
Mole, H. F.—Report on surgery, 243.
Morton, C. A.—Report on surgery, 157.

Nature, The healing powers of—G. M. Smith, 321.
Nixon, Dr. J. A.—Report on dermatology, 161; Thomas Dover: physician and merchant adventurer, 31.
Nose, Congenital deformity of, and results of subcutaneous injection of wax—Dr. E. H. E. Stack, 119.

Obstetrics, Report on — D. C. Rayner, 343.
Otitis media purulenta, Some points in the technique of the radical operation for chronic—Dr. P. W. Williams, 224.
Otology, Report on—Dr. P. W. Williams, 69.

Paraffin wax, Congenital deformity of nose treated by subcutaneous injection of—Dr. E. H. E. Stack, 119.
Parathyroids, Relation between calcium metabolism, tetany and the, 240.
Parker, Dr. G.—The causes of transient cerebral paralyses, 15; Report on Medicine, 339.
Parsons, Dr. J. H.—The Long Fox lecture on metastatic inflammations of the eye, 1.
Percussion notes, Theoretical considerations of pulmonary—Dr. G. H.-H. Almond, 215.
Pericolitis and diverticulitis, 68.
Pharynx, Abnormal pulsating vessels in the, 69.
Physique of boys, The—Dr. B. M. H. Rogers, 27.
Plantaris tendon, Rupture of, 187.
Pneumonia, Cases of—Dr. A. Fells, 336.
Pregnancy, Case of early, 91.
Puerperal sepsis, 344.
Puerperium, Cretinism and the—Dr. D. A. Alexander, 128.
Pulmonary percussion notes, Theoretical considerations of—Dr. G. H.-H. Almond, 215.

Quackery, The control of, 370.

Rayner, D. C.—Report on obstetrics, 343.
Rogers, Dr. B. M. H.—The physique of boys, 27.
Russell, E.—The adulteration of milk in Bristol, 47.

Schoolboys and long runs, 81.
Schools in Bristol, Medical inspection of, 274.
Sepsis treated with vaccines, 345.
Sick, Preparations for the—
  Aldoform, 375.
  Antineurasthin, 376.
  Arsan, 375.
  Arylarsonates, 86.
  Autan, 88.
  Biogene, 281.
  Bromoglidine, 281.
  Calomel cream, 85.
  Diamalt, 183.
  Digipuratum, 86.
  Elixir lactopeptine, 87.
  Fer ascoli, 375.
  Ferro-glidine, 375.
  Formenthoids, 375.
  Glidine, 183.
  Guaiacose, 88.
  Iodival, 86.
  Iodoglidine, 183.

Sick, Preparations for the (*cont.*)—
Ionic medication, Solid products for, 279.
Kephaldol, 376.
Kharsin, 87.
Lactic bacillary tablets, 280.
Lactilloids, 374.
Laxol, 281.
Luesan, 375.
Methylanodyne, 375.
Orsudan, 87.
Pulverettes, 86.
Roboleine, 86.
Sabromin, 280.
Sagrada barber, 280.
Soamin, 86.
Sumner's covers, 282.
Tabloid: Calcium iodo-ricinoleate, 280.
,, Hypodermic preparations, 87.
,, Laxative fruit pastilles, 280.
,, Pepana, 280.
,, Phenol and menthol compound, 280.
Tannismut, 88.
Thymoform, 375.
Thyresol, 281.
Tyramine, 374.
Vaporole: Cocaine hydrochloride, 279.
,, Morphine hydrochloride, 279.
,, Pituitary extract, 279
Veronal-sodium, 280.
Sleep and the modern hypnotics—Dr. J. M. Fortescue-Brickdale, 230.
Small-pox in Bristol and neighbourhood—Dr. D. S. Davies, 97.
Smith, Drs. I. W. Hall and W. A. —The value of some lactic acid ferments, 56.
Smith, G. M.—"Vis medicatrix naturæ," 321.
Society, Bristol Medico-Chirurgical, 91, 186, 378.
Sporotrichosis, 161.
Stack, Dr. E. H. E.—A family with a congenital deformity of the nose, and the results of subcutaneous injection of wax, 119.
Surgery in ancient and mediæval times, History and practice of—Dr. J. Swain, 289
Surgery, Reports on—E. W. H. Groves, 62; H. F. Mole, 243; C. A. Morton, 157; H. G. Kyle, 346.
Swain, Dr. J.—The history and practice of surgery in ancient and mediæval times, 289.

Tabes dorsalis, The mercurial treatment of—Dr. M. Faure, 312.
Tabetic ataxia, 276.
Tayler, Dr. H. P.—Contribution to discussion on treatment by ionisation or cataphoresis, 132; Electricity in general practice, 145.
Taylor, Resignation of Mr. J., 85.
Territorial R.A.M.C.—Formation of 2nd Southern General Hospital, 96; Progress of the 3rd South Midland Field Ambulance, 178; Presentation of drum-major's staff, 285.
Tetany and the parathyroids, Relation between calcium metabolism, 240.
Thomson, Dr. F. G.—Referred cardiac pain, 193.
Tuberculosis of the colon—E. W. H. Groves, 203.
Tuberculosis, The campaign against, 181.
Typhoid fever convalescents, 286.

Ulcerative colitis, Natural history of, 187.
University College Colston Society 78.
University of Bristol, 94, 178, 191, 272; Examination successes, 93, 189, 284, 383; Inaugural matriculation, 368.
Uterus, Fibromyomata of: occurrence of red degeneration in, 343.

Vaccines, Sepsis treated by, 345.
"Vis medicatrix naturæ"—G. M. Smith, 321.
Vomiting connected with anæsthesia —A. L. Flemming, 40.

Williams, Dr. P. W.—Report on laryngology and otology, 69; Some points in the technique of the radical operation for chronic otitis media purulenta, 224.

Zoophil-psychosis, The, 182.

J. W. Arrowsmith, Printer, Quay Street Bristol.

